# The PROCESS study: a protocol to evaluate the implementation, mechanisms of effect and context of an intervention to enhance public health centres in Tororo, Uganda

**DOI:** 10.1186/1748-5908-8-113

**Published:** 2013-09-30

**Authors:** Clare IR Chandler, Deborah DiLiberto, Susan Nayiga, Lilian Taaka, Christine Nabirye, Miriam Kayendeke, Eleanor Hutchinson, James Kizito, Catherine Maiteki-Sebuguzi, Moses R Kamya, Sarah G Staedke

**Affiliations:** 1 Department of Global Health and Development, London School of Hygiene & Tropical Medicine, 15-17 Tavistock Place, WC1H 9SH, London, UK; 2 Department of Clinical Research, London School of Hygiene & Tropical Medicine, WC1E 7HT, London, UK; 3 Infectious Disease Research Collaboration, PO Box 7475, Kampala, Uganda; 4 Department of Medicine, Makerere University, Kampala, Uganda

**Keywords:** Process evaluation, Theory-driven evaluation, Pathways of change, Complex interventions, Cluster randomised trial

## Abstract

**Background:**

Despite significant investments into health improvement programmes in Uganda, health indicators and access to healthcare remain poor across the country. The PRIME trial aims to evaluate the impact of a complex intervention delivered in public health centres on health outcomes of children and management of malaria in rural Uganda. The intervention consists of four components: Health Centre Management; Fever Case Management; Patient- Centered Services; and support for supplies of malaria diagnostics and antimalarial drugs.

**Methods:**

The PROCESS study will use mixed methods to evaluate the processes, mechanisms of change, and context of the PRIME intervention by addressing five objectives. First, to develop a comprehensive logic model of the intervention, articulating the project’s hypothesised pathways to trial outcomes. Second, to evaluate the implementation of the intervention, including health worker training, health centre management tools, and the supply of artemether-lumefantrine (AL) and rapid diagnostic tests (RDTs) for malaria. Third, to understand mechanisms of change of the intervention components, including testing hypotheses and interpreting realities of the intervention, including resistance, in context. Fourth, to develop a contextual record over time of factors that may have affected implementation of the intervention, mechanisms of change, and trial outcomes, including factors at population, health centre and district levels. Fifth, to capture broader expected and unexpected impacts of the intervention and trial activities among community members, health centre workers, and private providers. Methods will include intervention logic mapping, questionnaires, recorded consultations, in-depth interviews, focus group discussions, and contextual data documentation.

**Discussion:**

The findings of this PROCESS study will be interpreted alongside the PRIME trial results. This will enable a greater ability to generalise the findings of the main trial. The investigators will attempt to assess which methods are most informative in such evaluations of complex interventions in low-resource settings.

**Trial registration:**

Clinicaltrials.gov, NCT01024426

## Background

The majority of developing countries are not on target to achieve Millennium Development Goals four and five: to reduce the mortality rate of children under five by two-thirds and the maternal mortality ratio by three-quarters between 1990 and 2015 [1]. Malaria is a key focus for achieving these goals, with the latest World Health Organisation (WHO) guidelines promoting access to improved case management, including diagnostic testing for all suspected cases [2]. Failure to reach health targets has often been blamed on 'health system bottlenecks’ [1,3], typically cited as inadequate numbers, quality and distribution of health workers, equipment, supplies, and infrastructure [4]. In addition, some have argued that the way health services and programs are enacted in practice is a social as well as a structural issue; a function of interactions between clients, communities, health workers, and systems [5,6].

In spite of significant investments in Uganda since the early 1990s into programmes intended to improve health and access to quality healthcare, health outcomes remain poor across the country [7]. This poor progress has been attributed by some to a lack of alignment between externally defined programme priorities, and the priorities of local populations [8-12]. A more comprehensive approach to healthcare is called for to improve management of malaria and other febrile illnesses, attract patients to seek care, and produce health benefits at the population level.

Reviews of empirical research suggest that simple interventions to improve access to quality malaria care such as basic training or health education have had limited effect on changing healthcare provider behaviour [13,14] or the behaviour of populations [15]. Achieving a change in behaviour may require complex interventions that address the multiple factors involved with delivery of appropriate treatment [16,17]. After undertaking formative research in Tororo, eastern Uganda, to understand local realities and aspirations for quality of care, an intervention package was designed to improve access to good quality healthcare for the local population [18]. The PRIME intervention consists of four components: workshops in Health Centre Management for health workers in-charge of facilities; training and supervision visits in Fever Case Management for all health workers; workshops in Patient Centered Services for all health workers; and support of supply of artemether-lumefantrine (AL) and rapid diagnostic tests (RDTs) when stocks run low. Table 1 shows the topics for each module covered in the PRIME intervention. The manuals for delivering the intervention are available online at http://www.actconsortium.org.

**Table 1:** PRIME intervention components, modules and topics

**Intervention component**	**Module**	**Module title**	Topic***
**Health centre**	HCM 00*	Introduction to HCM	▪ Accountability
**Management (HCM)**	HCM 01*	Primary Healthcare (PHC) Fund management	▪ Budgeting and accounting using the PHC Fund management tool
			▪ Budgeting and accounting – putting it all together
	HCM 02	Drug Supply	▪ Principles of the drug distribution system
		Management	▪ Forms required in drug distribution cycle
			▪ The ACT Drug Distribution Assessment Tool (ADDAT)
	HCM 03	Health Information	▪ Why quality information matters
		Management	▪ The information cycle – from patient to patient
**Fever case management**	FCM-T	Fever case management training	▪ How to evaluate patients with fever and select patients for Rapid Diagnostic testing
**(FCM)**			▪ Performing and reading an RDT
			▪ Management of a patient with fever and a positive RDT
			▪ Management of a patient with fever and a negative RDT
			▪ Recognition and referral of patients with severe illness
			▪ Patient education
			▪ RDT storage and monitoring
	FCM-S	Supervision visits	▪ First supervision visit: within 1 week of training
			▪ Follow-up supervision visits: 6 weeks and 6 months after initial training
**Patient-centered**	PCS 00	Introduction to PCS	▪ Thinking about my role as a health worker
**Services (PCS)**			▪ Introduction to PCS
			▪ Introduction to Self Observation Activities
	PCS 01	Communication Skills	▪ Building Rapport
		Part 1	▪ Active listening
	PCS 02	Communication Skills	▪ Asking good questions
		Part 2	▪ Giving good information
	PCS 03	Building a positive work	▪ Health Centre Management Changes
		environment	▪ Dealing with stress at work
	PCS 04	Improving the Patient	▪ Communication Review
		Visit	▪ Patient Welcome and Orientation
	PCS 05**	Volunteers: Improving	▪ Patient Centres Services
		the Patient Visit	▪ Welcoming and greeting patients
			▪ Improving patient navigation

*These two modules are to be covered in the same workshop.

**This workshop was designed for anyone working or volunteering at health centers without medical training.

***For information on learning outcomes for each module, please see the summary of training and manuals online at http://www.actconsortium.org.

**Figure 1:**
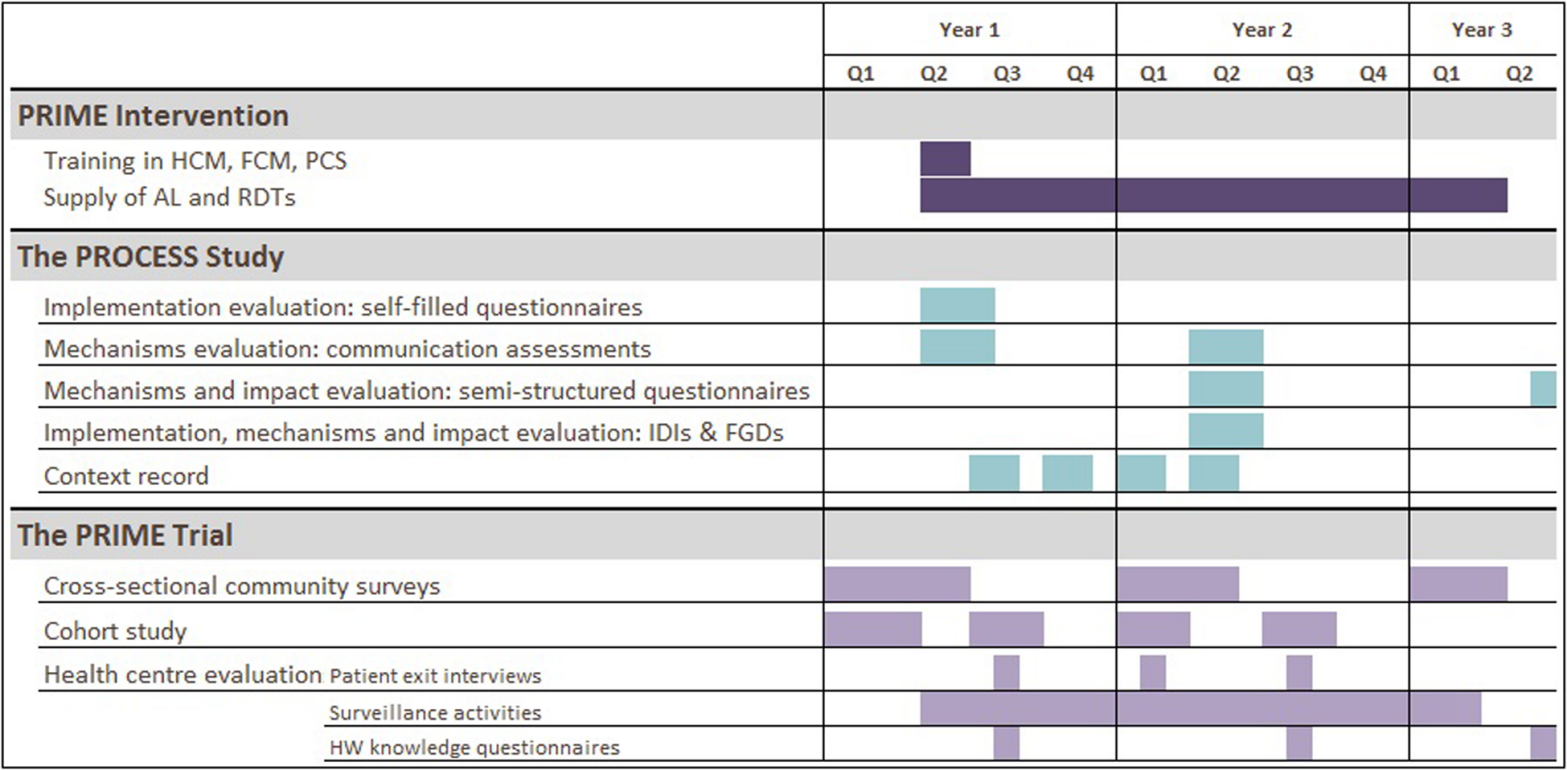
Timeline of activities for the ACT PROCESS study.

The primary and secondary outcomes of the PRIME intervention are being evaluated in a cluster-randomised trial comparing health centres that receive the intervention with 'standard care’ health centres that do not receive the intervention. The PRIME trial is evaluating outcomes on three levels: an annual cross-sectional community survey will assess the impact of the intervention on key population-based health indicators in children under fifteen, with the primary outcome as prevalence of anaemia in children under five; a cohort study will assess the impact of the intervention on key longitudinal indicators in children under five, with antimalarial treatment incidence density as the primary outcome; and patient exit interviews will assess the impact of the intervention on key indicators of case management for malaria and other illnesses in children under five treated at health centres, with the primary outcome as inappropriate treatment of malaria. The timing of the PRIME evaluation activities can be seen in Figure 1 and further details of the trial protocol can be found elsewhere [19].

Many researchers are now arguing for more comprehensive evaluations of complex interventions that attend to implementation, mechanisms of change, and context [20]. Such evaluations have been uncommon, and many of those that have existed alongside randomised controlled trials have been critiqued for poor integration with quantitative findings and methodological limitations [21], prompting the challenge for more carefully planned evaluations. In the PROCESS study, we adopt a theory-driven approach to our evaluation, aiming to understand what the PRIME intervention was and what it did, and aiming to contribute to broader discussions of how quality of care may be changed in similar contexts. It has been argued that carrying out theory-driven evaluations, including realist evaluation and the theory-of-change approach, is a way forward for opening the 'black box’ and exploring the modes of effects of complex interventions [22,23], including within randomised controlled trials [24]. There is much variation in the concerns and methods used by those adopting a theory-driven evaluation approach [25,26], although most propose to explicate a theory or model of the programme/intervention and use this to guide and strengthen evaluation questions and analysis [27]. We set out to understand the PRIME intervention and its actions by mapping out the intended intervention programme and contrasting this with the realities of implementation in practice and local interpretations of intervention effects, as well as interpreting contextual influences and attempting to assess impact within and outside of the intended consequences of the intervention.

## Methods and analysis

### Aim

The PROCESS study aims to evaluate the implementation, mechanisms of change and context of the PRIME intervention at health centres in rural Uganda to inform interpretation of outcomes in the main cluster-randomised trial.

### Objectives

1. To develop a comprehensive logic model of the PRIME intervention, mapping components of the intervention through to their intended effects and outcomes.

2. To evaluate the implementation of the intervention, including health worker training, health centre management tools, and the support of supply of AL and RDTs for malaria when stocks run low.

3. To understand mechanisms of change of the intervention components, including testing hypotheses and interpreting realities of intervention components in context.

4. To develop a contextual record over time of factors that may have affected intervention implementation, mechanisms of change and trial outcomes, including factors at community, health centre and district levels.

5. To capture broader expected and unexpected impacts of the intervention and trial activities in communities, public health centres, and at private providers.

### Study setting

The PRIME trial is taking place in seven sub-counties of Tororo District, eastern Uganda. Detailed descriptions of the health centres, population profile relating to health, and a history of interventions in the area can be found elsewhere [18,28]. All lower-level public health centres in the study area (n = 22) were eligible to participate, but due to overlapping catchment areas for two pairs of health centres, one from each pair was randomly excluded. A total of 10 health centres were then randomly allocated to receive the intervention and 10 to continue with standard care (n = 10). Further information about the randomisation process and other details of the main trial can be found in the trial protocol [19]. The area is highly endemic for malaria, with an estimated 562 infective bites per person per year [29]. The clinical focus of the intervention was therefore on the diagnosis and management of malaria.

### Study design

**Figure 2:**
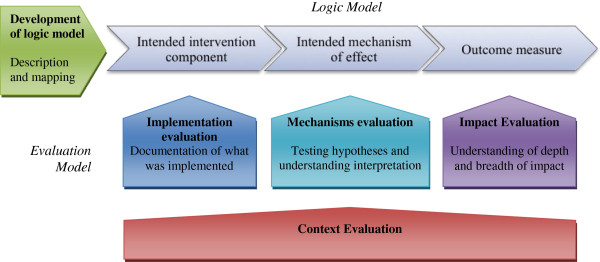
Framework for ACT PROCESS study.

The PROCESS study is a mixed-method evaluation. Figure 2 depicts our framework for the evaluation activities. Our focus in this study is on documenting the PRIME intervention, understanding the mechanisms involved in the PRIME intervention in practice, and describing the context of the PRIME intervention and evaluation. The majority of impact evaluation activities are being carried out under the PRIME trial [19].

### Objective one: development of a logic model

The articulation of an intervention’s intended pathway of change is recommended as a starting point for evaluation [30]. The output of this articulation is variously referred to as a 'change model’ [31], 'logic model’ [32], and 'theory of change’ [33]. Here, we use the term 'logic model’ because we see it as a display of the logic specific to this intervention trial rather than a theory that has broader application. The logic model for the PRIME intervention trial intends to articulate a set of hypotheses and assumptions upon which the trial’s outcomes and activities are based. The logic model is intended to set out the pathway of change from the PRIME intervention inputs through to the outcomes measured in the PRIME evaluation, incorporating proximal mechanisms and the conditions assumed to be required in order to support change. The development of the logic model is intended to be a process that can lead to refinement of intervention design as well as guide the design of evaluation activities [34]. Its development is therefore planned to be a dynamic process, occurring alongside intervention development, and involving team members involved in both the PRIME intervention and PRIME evaluation activities.

### Objective two: implementation evaluation

The implementation evaluation aims to document how the intervention is delivered and received, and to compare this with intended implementation. This should equip the research team to know whether trial outcomes can be attributed to the intended intervention or if there was implementation failure [22]. The intended implementation, consisting of inputs, process, and outputs—elsewhere termed 'implementation theory’ [23], 'process theory’ [35], or 'action model’ [31]—will be described elsewhere, including the rationale for the design. The implementation will be assessed in terms of the aspects detailed in Table 2, following Saunders *et al.*[36], for each of the PRIME training intervention components (Health Centre Management, Fever Case Management, and Patient- Centered Services).

**Table 2:** Implementation evaluation assessment domains, questions and methods

**Assessment domain**	**Questions relating to PRIME training**	**Data collection methods**
Fidelity	How much of the PRIME training was delivered as intended? What parts were not delivered?	Trainer questionnaires; direct observations
Reach	How much of the intended audience was exposed to the PRIME training?	Participant questionnaires
Dose delivered	What parts of the PRIME training were delivered most and least successfully to participants?	Trainer questionnaires; direct observations
Dose received	Which objectives, content and activities of the PRIME training were understood/absorbed best by participants?	Participant questionnaires; direct observations
Effectiveness	Did the training achieve its objectives according to proximal outcomes for participants?	Participant questionnaires
Recruitment	What procedures were necessary to encourage recruitment?	Trainer questionnaires; direct observations
Context	What social, logistical and political factors affected the delivery and receipt of the PRIME training?	Trainer questionnaires; direct observations; implementer, stakeholder and health worker in-depth interviews

### Self-filled questionnaires

Data will be collected through self-filled questionnaires completed by all trainers (n = 5) and participants (n = 40 – 50) after each workshop, for which written consent will be obtained at the first workshop. Questions will elicit yes/no responses, four-point Likert scale responses to statements from 'strongly agree’ to 'strongly disagree’ and open-ended responses. Participants will be encouraged to complete questionnaires honestly, with no repercussions for involvement with the project and with confidentiality assured. Observational data will also be collected in a non-structured format by the implementation evaluation team during trainings, to record any aspects relevant to fidelity of the implementation, dose delivered and received, recruitment, and contextual factors. Finally, in-depth interviews with implementers, stakeholders and health workers will be carried out 9 to 12 months after the training, with topic guides including questions relating to the implementation of activities. Analysis will involve descriptive statistics and content summaries of the open text fields in the questionnaires. In-depth interviews will be analysed thematically, using NVivo software to code the transcripts.

### Objective three: mechanisms evaluation

The mechanisms evaluation aims to describe the 'actual’ pathways of change from intervention inputs to measured outcomes, enabling comparison with the intended pathways of change in the trial’s logic model. This will involve two lines of questioning: first, testing hypotheses from the logic model, evaluating whether intended mechanisms 'work’ as planned; and second, understanding the way that the intervention ideas, principles, and materials are taken up, adapted, contested, rejected, and recast in context.

### Hypothesis testing

The ability to test hypotheses within the intended pathways of change of an intervention trial is important for the trials such as PRIME where a randomised approach can be undermined by a long and complex pathway from the intervention inputs delivered to health workers and the trial outcomes at the population level [37]. Hypothesis testing enables the research team to establish plausibility that outcomes are attributable to the action of the intervention, rather than to other factors or a different mechanism triggered by the intervention [38,39]. Hypothesis testing also enables consideration that outcomes are attributable to particular aspects of the intervention.

### Semi-structured questionnaires

For each of the intervention components, questionnaires will be designed for self-completion by all health workers (n ≅ 60) and all health workers in-charge (n ≅ 20) in both arms of the trial between 9 and 12 months after the intervention started. The questionnaires will attempt to assess change in line with each intended output of the intervention through the comparison between arms of a series of responses to statements with four-point Likert scale response options, from 'strongly agree’ to 'strongly disagree.’ Respondents will be asked to provide written consent at the start of the questionnaire. Open-ended questions will also be included on each topic to encourage expansion by respondents. Questions to assess the Health Centre Management component will ask for health worker in-charge experiences and opinions on financial management, stock management, and health management information systems. Questions to assess the proximal impact of Patient- Centred Services, will be designed to assess 'patient-centeredness,’ adapting a tool tested in several Northern countries [40]. An assessment of health worker motivation and feelings towards work will be undertaken within the questionnaire by adapting a tool piloted in Tanzania [41], generating scores for 'internal motivation’ and 'external motivation.’ Questions about malaria case management will cover knowledge, confidence, trust in tests, and other factors that may hinder or support quality case management. For each domain, aggregate scores will be created and will be compared between arms using one-way analysis of variance. Clustering of responses by health centre will be assessed through the intra-class correlation coefficient and will be adjusted for in the analysis if significant (ρ >0.1) using a random effects linear regression model in STATA (Statacorp, Texas). Finally, health workers in the intervention arm only will be asked to reflect on the usefulness of the training they received from PRIME and the frequency they used the information and skills learned. Descriptive statistics will be used to report on responses to each question.

### Communication assessments

A key hypothesis in the PRIME intended pathway of change is the improvement of health worker communication due to participation in the Patient-Centered Services workshops, and the impact of this on patient or caregiver satisfaction with the consultation, specifically with the interpersonal skills of the health worker. We will attempt to assess both the patient-centeredness of health worker communication in both arms, and the satisfaction of a subset of caregivers of children under five in both arms. This will first involve the audio recording of consultations, which will be rated for patient-centeredness following domains and rating methods developed by the Patient-Doctor Communication Group in Canada [42,43]. Second, caregivers will be interviewed on exit from the consultation and asked a series of questions following the same domains as the audio recording rating, with questions adapted from an existing questionnaire also developed in Canada [44]. The questionnaire will be translated and pretested. Responses will be allocated an aggregate score for each domain, following the format of the recorded consultations. The target sample size is 100 consultations and exit interviews, spread equally across the 10 intervention and 10 standard care health centres. Both health workers and caregivers will be informed about the study and asked to give written consent before participating. We will repeat this communication assessment three times: at baseline, immediately after the intervention training, and between 9 and 12 months after the intervention. At each time point, the same health workers will be sought out to participate to enable within-subject consistency. Comparisons of scores for the consultations and exit interviews will be drawn between arms using the same methods as for the semi-structured questionnaires described above.

### Enactment of the intervention

The ability to understand how the intervention was interpreted and enacted by different actors is especially important in cluster trials with relatively few units randomised to each arm; 10 in each for the PRIME trial. Here there is potential for large between-cluster differences in outcomes and receptiveness to interventions, which limits interpretation of results of cluster randomised trials [37]. An in-depth understanding of the context into which the intervention is introduced, and of the interpretations of the intervention by actors expected to make changes is important to learn what was useful about the intervention, for whom, and why. This should enable more meaningful interpretation of the generalisability of the intervention to different settings [24].

In our endeavour to understand how the intervention is enacted, we are concerned with the meanings it presents to different actors, drawing on an interpretativist perspective [45] as well as with the socio-material network of resources, forms, people, and groups that enact the intervention, drawing on a relational perspective [46]. While we are inspired by the premise of realist evaluation, with an aim to understanding 'what works, for whom, in what circumstances, and why?’ [47], we do not follow closely the methodology of interrogating many potential Context-Mechanism-Outcome triads, which assume each are bounded entities. Rather, we see the intervention as a script that is produced or reproduced in different activities and interactions, generating meaning in an ongoing sense for actors engaging with it. Our focus in this part of the PROCESS evaluation is therefore to understand the socio-material elements that constitute change, including but not limited to the methods, contents, and materials of the PRIME intervention.

### In-depth interviews

We will carry out in-depth interviews with a range of actors to elicit narratives of the intervention and its objectives in context, including its effects and factors shaping interpretation and integration of its ideas, resources, and processes. We will invite one health worker per intervention health centre to be interviewed (n = 10), as well as a range of sub-county and district representatives from across the study area (n = 10). We will also interview implementers of the PRIME intervention including trainers and those involved in delivering supplies (n = 6). Interviews will be tape-recorded, transcribed, and translated where necessary. Transcripts will be imported into NVivo version 8 (QSR International) and coded iteratively: ideas emerging will be labelled and grouped into themes as patterns emerge. Particular attention will be paid to understanding what and how changes are perceived to have occurred as well as analysis of the way each intervention was taken up, or not, in practice.

### Focus group discussions

To understand interpretations by community members of the PRIME intervention, which intended to impact health outcomes through enhancing health centres, we will carry out a series of focus group discussions (FGDs). Two target groups from populations in the study area will be invited: primary caregivers, representing those most frequently visiting public health centres; and household heads, representing those with influence over family resources as well as those privy to local political discourses. Three further sub-groups will be included according to location: FGDs will be held with groups of participants living in close proximity (within a two-kilometre radius) of intervention health centres; in close proximity to standard care health centres; or outside of a two-kilometre radius of either intervention or standard care health centres. We aim to carry out 12 FGDs following the matrix shown in Table 3. Participants will be invited in advance, selected with the help of local leaders, and will be asked to give written consent prior to the start of the FGD. Discussions will be tape-recorded and field notes will be taken. Transcription, translation, coding, and analysis will take place as for the in-depth interviews.

**Table 3:** Sampling matrix for number of community focus group discussions

	**Live close (<2 km) to intervention health centres**	**Live close (<2 km) to standard care health centres**	**Live away (>2 km) from either intervention or standard care facilities**
Primary care givers	3	3	3
Household heads	1	1	1

### Objective four: context record

The context record aims to document information relating to the context of the trial that may have affected the intervention’s implementation, mechanisms of change, and the outcomes under measurement. A data collection exercise will be undertaken every three months, starting before the intervention and ending after one year (time points = 5), to record information sources at three levels: district health officials (n = 5 – 10); health centre staff (n = 20); and community representatives (n = 10 – 15, including health assistants and lay key informants from each sub-county). At each time point, respondents will be asked a series of structured and open questions to elicit information about the past three months in terms of any (non-trial) activities, events, policies, infrastructure, human resources, media stories, environmental or other changes that have occurred that might have impacted: health workers’ ability to engage with the PRIME intervention and to provide quality care; patient and caregivers’ ability or willingness to access care at public health centres or elsewhere; and health outcomes at the population level. The data will be typed into Microsoft Word and reviewed to extract relevant information into a timeline in Microsoft Excel. Activities/events will be colour-coded into the following categories: resources; information/education/communication; organisation/policy; and other. The timeline will be available to consult when results from other sources (both quantitative and qualitative) begin to emerge, in order to understand patterns appearing in those data over time and between health centres and catchment areas.

### Objective five: impact evaluation

The impact evaluation component of this PROCESS study will be a small-scale undertaking, with the aim to understand some of the unintended impacts of the intervention [48]. The main PRIME trial will evaluate the impact of the intervention on community health outcomes. This PROCESS protocol aims to widen the lens to evaluate the impact of the trial on health centres, health workers, other providers, and community members. We will add questions to existing data collection activities listed above as well as conducting additional data collection with private providers.

### Most significant change method

The semi-structured questionnaires, in-depth interviews, and FGDs will begin with open questions about the most significant change participants observed in the way they worked/sought treatment, why this was significant, and what difference it has made to them. This method aims to collect and analyse systematically significant changes from the perspectives of those involved in a programme [49]. We will use this method to elicit broader responses from participants, particularly stories in the in-depth interviews and FGDs, before prompting for intended changes and pre-defined indicators in the methods outlined above.

### Private provider questionnaires

The landscape of treatment seeking in this context includes availability of antimalarials at private providers—registered and unregistered shops and clinics. An understanding of the role these providers play and the drugs and diagnostic abilities they have available is proposed to assist our understanding of the pathways of change and outcomes, as well as unintended impacts of the trial. We will therefore carry out a mapping exercise and interview private providers using a semi-structured questionnaire initially with 10 providers 9 to 12 months after the intervention and then with all providers at drug shops identified a year later, once subsidised ACTs become available through the Affordable Medicines Facility malaria (AMFm) mechanism in Uganda.

### Quality assurance

The study team will be trained in the project objectives, collection of high quality data [50], and good clinical practice (GCP) guidelines. Study personnel will be trained and mentored to maintain principles for good quality research practice throughout the research process, from the design of tools, to field work engagements, to data management and analysis [51]. The team will work together to devise, pilot and revise standard operating procedures (SOPs) for all study activities. These SOPs will be adhered to or adapted throughout the research process. Meetings will be held at least weekly between the field team and investigators to identify, discuss, and resolve any issues arising from the evaluation practice and study findings. Clear line management will be established within the study team, and frequent performance feedback will be given by the study investigators to the team leader and on to the members of the study team.

### Ethics

The study protocol and information sheets have been approved by the Ugandan National Council for Science and Technology (UNCST Ref HS 864), the Makerere University School of Medicine Research & Ethics Committee (SOMREC Ref 2011–103), and the London School of Hygiene and Tropical Medicine Ethics Committee (LSHTM Ref 5831). All potential participants will be informed of the purpose and nature of the study before being invited to participate and sign written consent forms. The discussion, information sheets and consent forms will be in the most appropriate language for the participant—whether English, Luganda, Japadhola, or Swahili—and a copy of the forms will be left with the participant. Risks and benefits of participation will be discussed and any questions relating to the research answered. Participants will be informed that all records will be kept as confidential as possible, with participants being recorded and quoted by number rather than name. However, participants will be made aware that any participation in the research study may involve a loss of privacy. Participants will be given the option of not being quoted at all, anonymously or otherwise, or included in any of the analyses. If a potential participant is unable to read or write, their fingerprint will substitute for a signature, together with a signature from a witness to the informed consent procedures.

## Trial status

The PRIME trial field work completed in July 2013. Data cleaning and analysis of the final community survey, including the primary outcome for the survey and the overall trial, has not yet begun. The PROCESS study field work completed in July 2013. Data analysis for the PROCESS study has been ongoing, in order to inform the development of lines of enquiry throughout the trial period.

## Discussion

The findings of this PROCESS study will be interpreted alongside the PRIME study. We plan to use the MATRICS (Method for Aggregating The Reporting of Interventions in Complex Studies) approach to bring together complex data from multiple sources to evaluate a complex intervention [52]. This evaluation represents a significant undertaking in addition to the main trial. The investigators will attempt to assess which methods are most informative in such evaluations of complex interventions in low-resource settings.

## Abbreviations

ACT: Artemisinin-based combination therapy; ADDAT: ACT Drug Distribution Assessment Tool; AL: Artemether-lumefantrine; FCM: Fever case management; HC: Health centre; HCM: Health centre management; HMIS: Health management information systems; LSHTM: London School of Hygiene and Tropical Medicine; PCS: Patient-centered services; PHC: Primary healthcare; RDT: Rapid diagnostic test; SOMREC: School of Medicine Research and Ethical Committee (Makerere University); UNCST: Uganda National Council of Science and Technology; WHO: World Health Organization.

## Competing interests

The authors declare that they have no competing interests.

## Authors’ contributions

CIRC and SGS conceived of the study and drafted the protocol with DD and SN. All authors contributed to the protocol, reviewed this manuscript and gave permission for publication. All authors read and approved the final manuscript.

## References

[B1] Lozano R Wang H Foreman KJ Progress towards millennium development goals 4 and 5 on maternal and child mortality: an updated systematic analysis Lancet 2011 378 9797 1139 1165 doi:10.1016/S0140-6736(11)61337-8 [published Online First: Epub Date] 10.1016/S0140-6736(11)61337-8 21937100

[B2] World Health Organisation Guidelines for the Treatment of Malaria Second Geneva Available online at http://www.who.int/malaria/publications/atoz/9789241547925/en/index.html 2010

[B3] Reich MR Takemi K Roberts MJ Global action on health systems: a proposal for the toyako G8 summit Lancet 2008 371 9615 865 869 doi:10.1016/S0140-6736(08)60384-0[published Online First: Epub Date]| 10.1016/S0140-6736(08)60384-0 18328932

[B4] Bhutta ZA Chopra M Axelson H Countdown to 2015 decade report (2000–10): taking stock of maternal, newborn, and child survival Lancet 2010 375 2032 2044 doi:10.1016/S0140-6736(10)60678-2 [published Online First: Epub Date] 10.1016/S0140-6736(10)60678-2 20569843

[B5] Bloom G Standing H Future health systems: Why future? Why now? Soc Sci Med 2008 66 10 2067 2075 doi:10.1016/j.socscimed.2008.01.032[published Online First: Epub Date] 10.1016/j.socscimed.2008.01.032 18321628

[B6] Mackian S Bedri N Lovel H Up the garden path and over the edge: where might health-seeking behaviour take us? Health Policy Plan 2004 19 3 137 146 10.1093/heapol/czh017 15070862

[B7] Kiwanuka SN Ekirapa EK Peterson S Access to and utilisation of health services for the poor in Uganda: a systematic review of available evidence Trans R Soc Trop Med Hyg 2008 102 11 1067 1074 doi:10.1016/j.trstmh.2008.04.023 [published Online First: Epub Date] 10.1016/j.trstmh.2008.04.023 18565559

[B8] Gonzaga B Kiyaga JN Reynolds Whyte S Health System Profile: Decentralisation of the Health Care System. A Study of Tororo and Busia Districts 1999 Kampala, Uganda: Tororo Community Health (TORCH) Project Online at http://www.chdc.mak.ac.ug/publications/Busulwa%20Gonzzaga%201999_Health%20Systems%20Profile%20Decentralisation%20of%20the%20Health%20Care%20System.pdf

[B9] Jitta J Reynolds Whyte S Nshakira N The availability of drugs: what does it mean in Ugandan primary care Health Policy 2003 65 2 167 179 10.1016/S0168-8510(03)00003-4 12849915

[B10] Kyaddondo D Whyte SR Working in a decentralized system: a threat to health workers' respect and survival in Uganda Int J Health Plann Manag 2003 18 4 329 342 10.1002/hpm.730 14727711

[B11] Mogensen HO Finding a path through the health unit: practical experience of Ugandan patients Med Anthropol 2005 24 3 209 236 doi:10.1080/01459740500182659 [published Online First: Epub Date] 10.1080/01459740500182659 16081334

[B12] Mutumba A The effect of decentralisation on the performance of district personnel in Uganda. A case-study of Tororo district health directorate 2005 Kampala: Makerere University

[B13] Grimshaw J Shirran L Thomas R Haines A, Donald A Changing provider behaviour: an overview of systematic reviews of interventions to promote implementation of research findings by healthcare professionals Getting Research Findings into Practice 2002 2 London: BMJ Books 29 68

[B14] Oxman AD Thomson MA Davis DA No magic bullets: a systematic review of 102 trials of interventions to improve professional practice Cmaj 1995 153 10 1423 1431 7585368 PMC1487455

[B15] Smith LA Jones C Meek S Review: provider practice and user behavior interventions to improve prompt and effective treatment of malaria: do we know what works? Am J Trop Med Hyg 2009 80 3 326 335 19270276

[B16] Ross-Degnan D Laing R Santoso B Improving pharmaceutical use in primary care in developing counties: a critical review of experience and lack of experience 1997 Chiang Mai, Thailand: Presented at the International Conference on Improving Use of Medicines

[B17] Power R Langhaug LF Nyamurera T Developing complex interventions for rigorous evaluation--a case study from rural Zimbabwe Health Educ Res 2004 19 5 570 575 doi:10.1093/her/cyg073 cyg073 10.1093/her/cyg073 15155588

[B18] Chandler CI Kizito J Taaka L Aspirations for quality health care in Uganda: How do we get there? Hum Resour Health 2013 11 1 13 doi:10.1186/1478-4491-11-13 [published Online First: Epub Date] 10.1186/1478-4491-11-13 23521859 PMC3610284

[B19] Staedke SG Chandler CIR Diliberto D The PRIME study protocol: evaluating the impact of an intervention implemented in public health centres on management of malaria and health outcomes of children using a cluster-randomised design in Tororo, Uganda Implement Sci 2013 8 1 114 10.1186/1748-5908-8-114 24079295 PMC3851935

[B20] MRC Developing and Evaluating Complex Interventions: new guidance London: Medical Research Council Available online at http://www.mrc.ac.uk/Utilities/Documentrecord/index.htm?d=MRC004871

[B21] Lewin S Glenton C Oxman AD Use of qualitative methods alongside randomised controlled trials of complex healthcare interventions: methodological study BMJ 2009 339 b3496 10.1136/bmj.b3496 19744976 PMC2741564

[B22] Chen H Rossi P Issues in the theory-driven perspective Eval Program Plann 1989 12 4 299 306 10.1016/0149-7189(89)90046-3

[B23] Weiss CH Connell JP, Kubisch AC, Schorr LB Nothing as Practical as Good Theory: exploring theory-based evaluation for comprehensive community initiatives for children and families New Approaches to Evaluating Community Initiatives: Volume 1, Concepts, Methods and Contexts 1995 Washington, DC: The Aspen Institute

[B24] Bonell C Fletcher A Morton M Realist randomised controlled trials: a new approach to evaluating complex public health interventions Soc Sci Med 2012 75 12 2299 2306 doi:10.1016/j.socscimed.2012.08.032 [published Online First: Epub Date] 10.1016/j.socscimed.2012.08.032 22989491

[B25] Marchal B van Belle S van Olmen J Is realist evaluation keeping its promise? A review of published empirical studies in the field of health systems research Evaluation 2012 18 2 192 212 doi:10.1177/1356389012442444 [published Online First: Epub Date] 10.1177/1356389012442444

[B26] Stame N Theory-based evaluation and types of complexity Evaluation 2004 10 1 58 76 doi:10.1177/1356389004043135 [published Online First: Epub Date] 10.1177/1356389004043135

[B27] Coryn CLS Noakes LA Westine CD A systematic review of theory-driven evaluation practice from 1990 to 2009 Am J Eval 2011 32 2 199 226 10.1177/1098214010389321

[B28] Staedke SG Phase 1 Report: Tororo District Survey Project. Characterizing the population and local health services 2010 Kampala, Uganda: Uganda Malaria Surveillance Project Online at http://www.actconsortium.org/data/files/actc_tororo_phase_i_report_final_10june10.pdf

[B29] OKELLO PE VAN BORTEL W BYARUHANGA AM Variation in malaria transmission intensity in seven sites throughout Uganda Am J Trop Med Hyg 2006 75 2 219 225 16896122

[B30] National Institute for Health and Clinical Excellence Behaviour Change at Population, Community and Individual Levels 2007 London: NICE Public Health Guidance

[B31] Chen HT Practical Program Evaluation. Assessing and improving planning, implementation, and effectiveness 2005 Thousand Oaks, CA: SAGE

[B32] Harris M Evaluating Public and Community Health Programs 2010 San Fransisco, CA: John Wiley & Sons

[B33] Mackenzie M Blamey A The practice and the theory: lessons from the application of a theories of change approach Evaluation 2005 11 2 151 168 doi:10.1177/1356389005055538 [published Online First: Epub Date] 10.1177/1356389005055538

[B34] Blamey AAM MacMillan F Fitzsimons CF Using programme theory to strengthen research protocol and intervention design within an RCT of a walking intervention Evaluation 2013 19 1 5 23 doi:10.1177/1356389012470681 [published Online First: Epub Date] 10.1177/1356389012470681

[B35] Donaldson SI Program theory-driven evaluation science 2007 New York, NY: Lawrence Erlbaum

[B36] Saunders RP Evans ME Joshi P Developing a process-evaluation plan for assessing health promotion program implementation: a how-to guide Health Promot Pract 2005 6 134 147 10.1177/1524839904273387 15855283

[B37] English M Schellenberg J Todd J Assessing health system interventions: key points when considering the value of randomization Bull World Health Organ 2011 89 12 907 912 doi:10.2471/BLT.11.089524 BLT.11.089524 10.2471/BLT.11.089524 22271948 PMC3260899

[B38] Habicht JP Victora CG Vaughan JP Evaluation designs for adequacy, plausibility and probability of public health programme performance and impact Int J Epidemiol 1999 28 1 10 18 10.1093/ije/28.1.10 10195658

[B39] Webster J Kweku M Dedzo M Evaluating delivery systems: complex evaluations and plausibility inference Am J Trop Med Hyg 2010 82 4 672 677 doi:10.4269/ajtmh.2010.09-0473 [published Online First: Epub Date] 10.4269/ajtmh.2010.09-0473 20348517 PMC2844570

[B40] Grol RP de Maeseneer J Whitfield M Disease-centred versus patient-centred attitudes: comparison of general practitioners in Belgium, Britain and The Netherlands Fam Pract 1990 7 2 100 103 10.1093/fampra/7.2.100 2369975

[B41] Chandler CIR Chonya S Mtei F Motivation, money and respect: a mixed-method study of Tanzanian non-physician clinicians Soc Sci Med 2009 68 11 2078 2088 doi:10.1016/j.socscimed.2009.03.007 [published Online First: Epub Date] 10.1016/j.socscimed.2009.03.007 19328607

[B42] Stewart M Brown JB Weston WW Patient-centered medicine: Transforming the clinical method 1995 Thousand Oaks, CA: Sage Publications

[B43] Brown JB Stewart MA Ryan BL Assessing communication between patients and physicians: The measure of patient-centred communication (MPCC). Working Paper Series, Paper # 95–2, 2nd Ed 2001 London, Ontario: Thames Valley Family Practice Research Unit and Centre for Studies in Family Medicine

[B44] Stewart M Meredith L Ryan BL The patient perception of patient-centredness questionnaire (PPPC): Centre for Studies in Family Medicine 2004 Working Paper Series #04-1 London, Ontario, Canada: The University of Western Ontario

[B45] Sobo EJ Culture and Meaning in Health Services Research 2009 Walnut Creek, CA: Left Coast Press

[B46] Koivisto J What evidence base? steps towards the relational evaluation of social interventions. Evidence & policy J Res Debate Prac 2007 3 527 537 0.1332/174426407782516529[published Online First: Epub Date] 10.1332/174426407782516529

[B47] Pawson R Nothing as practical as a good theory Evaluation 2003 9 4 471 490 doi:10.1177/1356389003094007 [published Online First: Epub Date] 10.1177/1356389003094007

[B48] Jones N Jones H Steer L Improving Impact Evaluation Production and Use: Overseas Development Institute 2009

[B49] Davies R Dart J The 'Most Significant Change’ (MSC) Technique. A Guide to Its Use 2005 Available online at: http://www.mande.co.uk/docs/MSCGuide.htm

[B50] Haaland A Molyneux CS Marsh V Quality information in field research: Training manual on practical communication skills for field researchers and project personnel: WHO/TDR 2006 Available online http://whqlibdoc.who.int/hq/2006/TDR_IRM_PCT_05.1_eng.pdf

[B51] Reynolds J Kizito J Ezumah N Quality assurance of qualitative research: a review of the discourse Health Res Policy Syst/ BioMed Central 2011 9 43 doi:10.1186/1478-4505-9-43 [published Online First: Epub Date] 10.1186/1478-4505-9-43 PMC3267652 22182674

[B52] Thorne K Jerzembek GS Cheung W MATRICS: A Method for Aggregating The Reporting of Interventions in Complex Studies. Clinical Trials Methodology Conference 2011 2011 Bristol, UK: Trials

